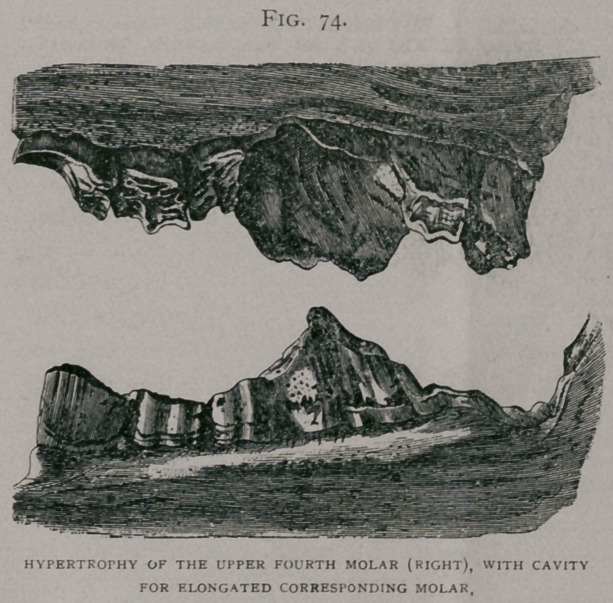# Age of the Horse, Ox, Dog, and Other Domesticated Animals

**Published:** 1891-06

**Authors:** R. S. Huidekoper

**Affiliations:** Vet.


### AGE OF THE HORSE, OX, DOG, AND OTHER DOMESTICATED ANIMALS.

BY R. S. HUIDEKOPER, M.D., VET.

[*Continued from page 231.*]

### MOLAR TEETH.

As in the case of the incisors the irregularities of the molar teeth are due to excess and deficiency of length. This may occur on one, or both sides of the jaw, on all of the tables of an arch, or only on certain teeth.

FIG. 71.

LOWER MOLAR ARCH OF A VERY OLD HORSE.

### DEFICIENCY OF LENGTH OF THE CROWN.

*Inferior Jaw.*—The abnormal use of the molars rarely extends to all of them in an arch ; sometimes those in the centre of the arch are the most worn (Fig. 71), giving it a concavity, in which the corresponding elongated teeth of the upper jaw fit ; or, sometimes they are worn most at the extremities of the arch, with an inverse condition of the upper jaw (Fig. 72) ; in molars which have been excessively worn the crown may have disappeared, leaving the roots as distinct and apparently supernumerary teeth (Fig. 71). In this case, as was noticed in the study of the structure of teeth, when they are worn down, they produce an irritation of their alveolar cavities, which causes an excessive deposit of calous bone or cement, which unites them together, and aids them in their function, but sometimes causes annoying bony tumors ; but the new function is not as complete as the original one, as the hard enamel has disappeared, and the wearing surface is much softer ; the mastication is not complete, and the animal shows the effect of a lessened nutrition.

*Superior Jaw.*—What has been said of the lower jaw applies to the upper, but the changes in them are less frequent. The separation of the roots in the upper jaw is less common, as these teeth are longer and contain a greater amount of enamel.

### EXCESS OF LENGTH OF THE CROWNS.

Excess of length of the crowns or portions of them is of rather common occurrence. As the upper molars are broader as well as longer than the lower ones, the lower jaw is obliged to have a lateral movement in order to grind against the whole table of the former. When the horse is fed under the ordinary conditions of nature, finding coarse weeds, twigs, hard vegetable matters and resisting substances, as dirt, etc., mixed with its food, this lateral movement is rendered complete by the slowness and difficulty of mastication, and the teeth are kept worn evenly. When, however, the animals are given selected fine hay, cleaned oats and slops and mashes, easy of mastication, they use the jaws like a chopping machine, and all parts of the grinding surfaces are not brought into contact. Again, when horses, like cab horses, doctors' hacks, etc., are fed at irregular times and in a hurry, they masticate incompletely, give the jaws but little lateral movement, and the narrower, lower jaws wear only against the internal portion of the tables of the upper teeth. When from any cause, such as caries of a tooth, cementoma, disease of any kind or deformity, the animal chews

FIG. 72.

MOLAR ARCHES (RIGHT) OF A VERY OLD HORSE.
Lower jaw to the left of figure.

more on one side than the other, the normal bevel of the teeth, throws the wearing surfaces from their natural position and while some portions are worn faster, others do not receive the proper amount of use. When one or more teeth have been partially destroyed or lost, the opposing teeth, finding lessened resistance, become excessive in length, and frequently irregular in shape. The irregularity of shape from any of the above causes is usually in the form of an increased bevel; only the internal surface of the upper molars is worn, causing the formation of a little ledge along their outer borders, this acts as a check to lateral motion of the lower jaw, soon prevents it altogether, and cause and effect soon react on each other. The external borders of the upper jaw become long, thin, and sharp, and their irregular ragged edges cut the cheeks and serve as lodging places for balls of food, which may decompose, causing further irritation to the mucous membranes The internal borders of the lower teeth may become very long and stand up like so many arrow points, cutting the tongue, and even penetrating the hard palate. The pressure on the border of the teeth worn close to their roots, and the lateral pressure due to the leverage of the crown on its root, in the alveolar cavity, produces further irritation and prevents proper mastication; the pain on one side frequently confines the grinding entirely to the other. The loss of the lateral motion also prevents the slight fore and back play of the arches of the teeth, and soon the anterior edge of the first molar above, and the

FIG. 73.

IRREGULAR WEARING OF UPPER AND LOWER MOLARS.

posterior edge of the last molar below, grow into points, which may be very annoying to the animal; removal of the latter is troublesome to the surgeon. Disease of a molar in one jaw, or its absence from fracture or otherwise, is soon followed by complication in the opposing tooth of the other jaw. Fig. 74 shows a fourth molar in the upper jaw with a cavity which was the size of a hen's egg, into which points the fourth inferior molar (elongated). The presence of a dental cyst probably explains the enlargement of the upper tooth and also the softness of its texture, which allowed it to be worn hollow.

FIG. 74.

HYPERTROPHY OF THE UPPER FOURTH MOLAR (RIGHT), WITH CAVITY FOR ELONGATED CORRESPONDING MOLAR.

Irregularity and deformity of the molars is the cause of much constitutional trouble; the interference with the mechanical action of the jaws, and the soreness produced in them, and in the cheeks and tongue renders trituritión of the food and mixture with saliva incomplete, the unprepared food is not digested and assimilated properly, causing a defective nutrition, the animal falls away in flesh, becomes hide bound, and may have attacks of indigestion. The non assimilatiou of the food causes indigestions and atony of the digestive tract and predisposes to intestinal calculi. The molars of all stable-fed animals should be looked to once or twice a year, including those of race colts which are grain fed from weaning. The inspection or the molars should constitute a part of examination for soundness.

[TO BE CONTINUED.]

## Figures and Tables

**Fig. 71. f1:**
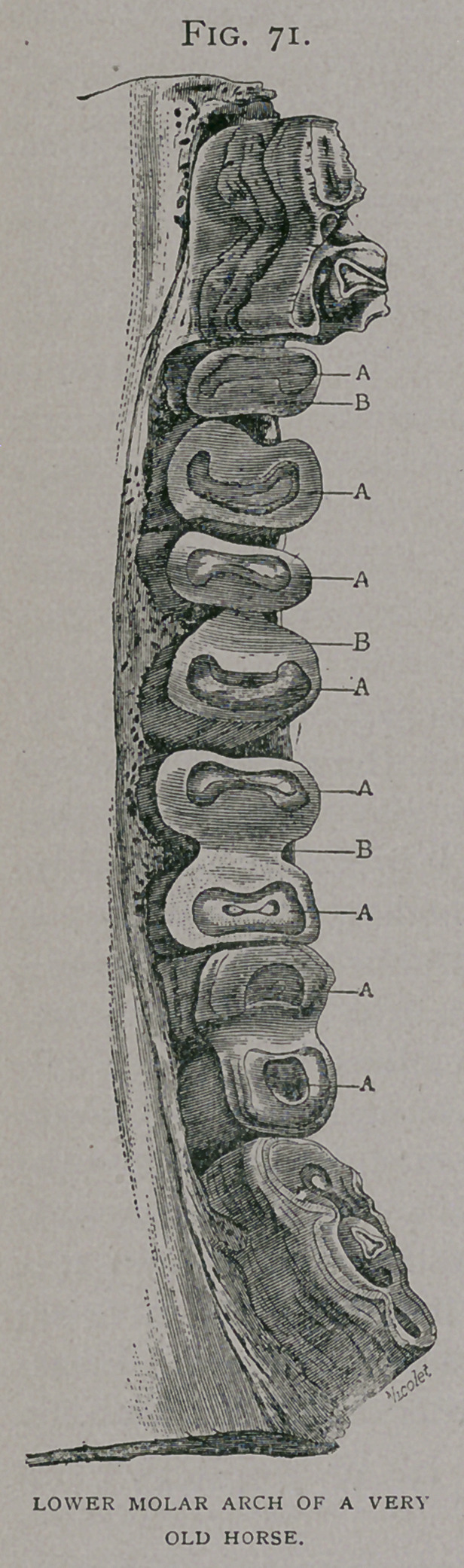


**Fig. 72. f2:**
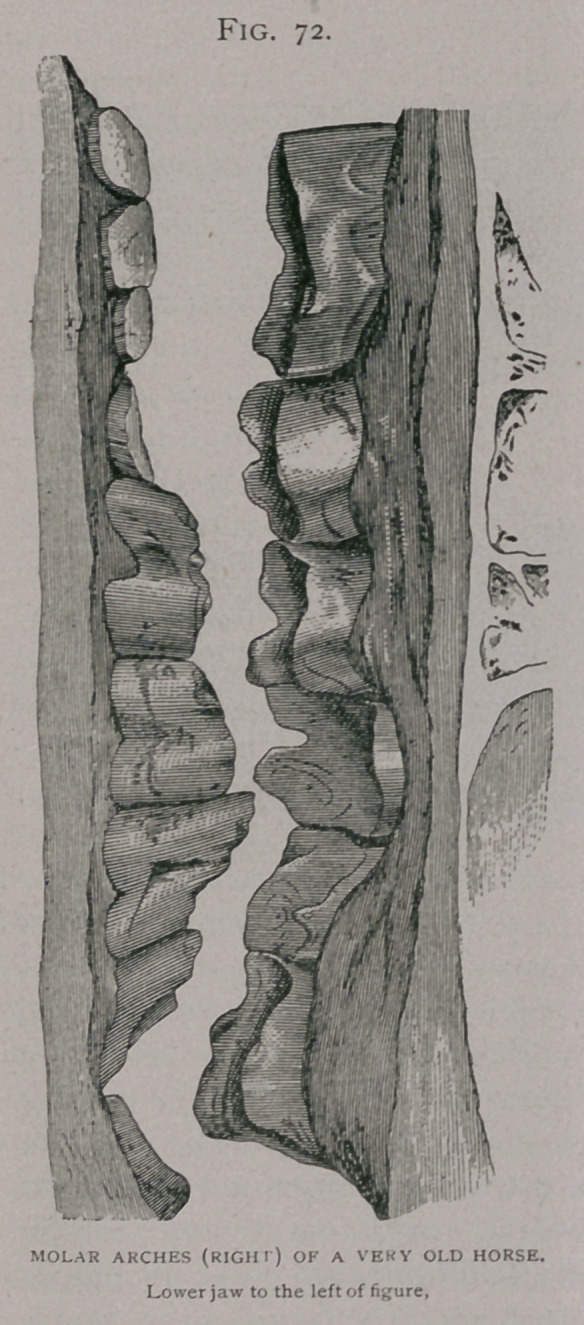


**Fig. 73. f3:**
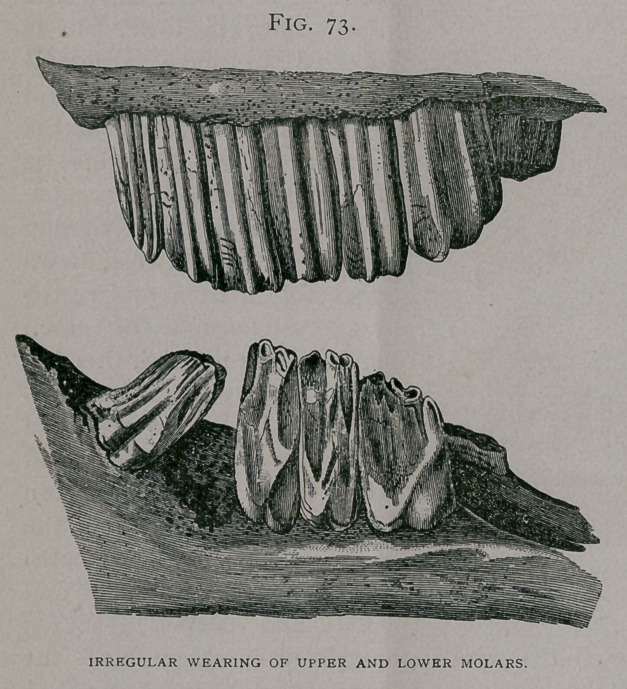


**Fig. 74. f4:**